# (‒)-Cannabidiolic Acid, a Still Overlooked Bioactive Compound: An Introductory Review and Preliminary Research

**DOI:** 10.3390/molecules25112638

**Published:** 2020-06-05

**Authors:** Marialuisa Formato, Giuseppina Crescente, Monica Scognamiglio, Antonio Fiorentino, Maria Tommasina Pecoraro, Simona Piccolella, Michelina Catauro, Severina Pacifico

**Affiliations:** 1 Department of Environmental, Biological and Pharmaceutical Sciences and Technologies, University of Campania “Luigi Vanvitelli”, Via Vivaldi 43, 81100 Caserta, Italy; marialuisa.formato@unicampania.it (M.F.); giuseppina.crescente@unicampania.it (G.C.); monica.scognamiglio@unicampania.it (M.S.); antonio.fiorentino@unicampania.it (A.F.); mariatommasina.pecoraro@unicampania.it (M.T.P.); simona.piccolella@unicampania.it (S.P.); 2 Department of Engineering, University of Campania “Luigi Vanvitelli”, Via Roma 29, I-81031 Aversa, Italy; michelina.catauro@unicampania.it

**Keywords:** cannabidiolic acid, *Cannabis sativa* L., hemp pollen, spectroscopic techniques, mass spectrometric techniques

## Abstract

Cannabidiolic acid (CBDA) is the main phytocannabinoid in fiber and seed-oil hemp (*Cannabis sativa* L.) plants, but its potential health-related capabilities have been masked for years by a greater scientific interest towards its neutral derivative cannabidiol (CBD). This review aims to collect from the literature and critically discuss all the information about this molecule, starting from its biosynthesis, and focusing on its bioactivity, as an anti-inflammatory, anti-emetic, anti-convulsant, and anti-cancerogenic drug. Furthermore, in the awareness that, despite its multiple bioactive effects, currently poor efforts have been made to achieve its reliable purification, herein, we propose a relatively simple, fast, and inexpensive procedure for its recovery from pollen of industrial hemp cultivars. Spectroscopic and spectrometric techniques allowed us to unequivocally identify pure isolated CBDA and to distinguish it from the constitutional isomer tetrahydrocannabinolic acid (THCA-A).

## 1. Introduction

The renewal of interest in industrial hemp (*Cannabis sativa* L.) launched new scientific research goals worldwide for its therapeutic, nutraceutical, and food applications [1,2,3]. Over time, hemp is reacquiring its role as green and sustainable crop, able to be a good alternative and ingredients resource for health foods, organic body care, biomaterials, and more [4]. Indeed, the hemp growing industrial market cannot disregard its diversity in chemical compounds, among which phytocannabinoids are the most described. These secondary metabolites phytochemically praise the uniqueness of *Cannabis* species, but also, in a puzzling scenario, dictated the denial of the plant use for almost 40 years of our history. The psychoactive/psychotropic Δ^9^-tetrahydrocannabinol (Δ^9^-THC or simply THC) was widely condemned as the guilty substance; nowadays, it is recognized as CB1 and CB2 partial agonist, whereas another abundant cannabinoid, namely cannabidiol (CBD), is identified as an “entourage compound”, able to modulate THC effects [5]. Indeed, *Cannabis* plant produces cannabinoids as prenylated aromatic carboxylic acids, which are converted in their more discussed neutral forms thanks to light, heat, or prolonged storage. In particular, on the basis of the concentrations of the main acidic cannabinoids, five *Cannabis* chemotypes occur. Chemotype I consist in drug-type plants for which the tetrahydrocannabinolic acid (THCA)/ cannabidiolic acid (CBDA) ratio is >>1.0, chemotype III describes plants whose aerial parts contain a THCA/CBDA ratio <<1.0, whereas an intermediate ratio corresponds to chemotype II. The high content of cannabigerolic acid (CBGA) is related to chemotype IV, and finally, chemotype V designates all the fiber-plants that completely lack cannabinoids [6].

The cultivation of industrial hemp varieties, which are approved by European Union, properly listed and with limited THC content, is augmenting the interest in the acidic derivatives. Cannabidiolic acid, which represents the main compound in fiber and seed-oil plants [7], is becoming a true protagonist, but its beneficial outcomes are still hidden and unexplored. Herein, the current knowledge on this overlooked molecule, whose bioactivity did not correspond to any great efforts to achieve its reliable purification, is briefly reviewed. Cannabidiolic acid (CBDA), which seems to share some several pharmacological features with its neutral analogue, could be favorably recovered by industrial hemp processing, and by the huge amount of its by-products and wastes.

## 2. CBDA and Its Biosynthesis

Cannabidiolic acid is a 22-carbon terpenophenolic compound and represents the main phytocannabinoid in the fiber and seed-oil hemp varieties [8]. The biosynthesis of this compound is through cannabidiolic acid synthase (CBDAS), a covalently flavinylated oxidase, which catalyzes the stereoselective oxidocyclization of cannabigerolic acid into CBDA [9].

**Figure 1:**
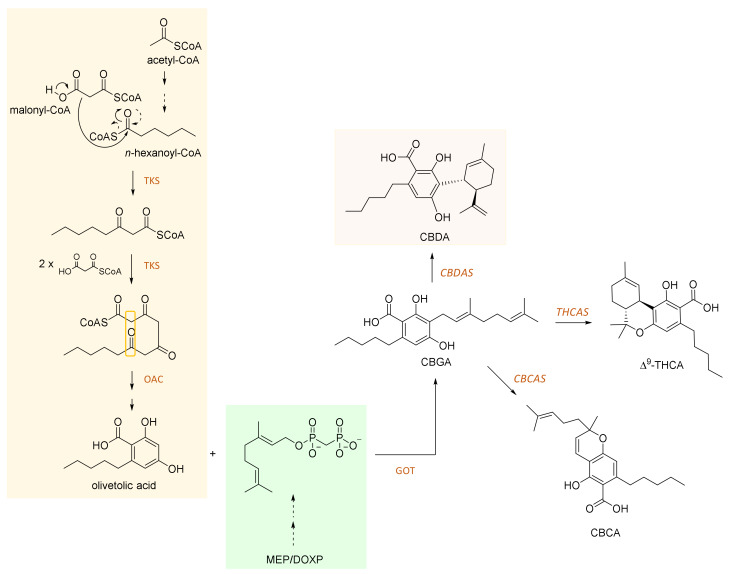
Schematic pathway for CBDA, THCA, and CBCA biosynthesis. Tetraketide synthase (TKS) catalyzes Claisen-like condensation between *n*-hexanoyl-CoA and three nucleophilic malonyl-CoA. The tetraketide obtained undergoes, through olivetolic acid cyclase (OAC), a polyketide cyclase enzyme, aldol-like condensation to achieve, following hydrolysis, olivetolic acid. The *C*-geranylation of the alkylresorcinolic acid by prenylase geranyl-diphosphate/olivetolate geranyl transferase (GOT) provides cannabigerolic acid (CBGA), which is converted by specific oxidocyclases into CBDA, THCA, and CBCA. These latter compounds undergo non-enzymatic conversion for achieving their relative neutral forms and/or other cannabinoid compounds.

CBDAS is an ancestral type from which tetrahydrocannabinolic acid synthase (THCAS) evolved [10,11,12]. Both CBDAS and THCAS belong to the family of berberine bridge enzyme-like enzymes [13]. Briefly, hexanoyl-CoA, which was found to be formed by an acyl-activating enzyme (AAE) in glandular trichomes [14], undergoes a Claisen-like condensation involving three malonyl-CoA nucleophiles. Thus, a tetra-β-ketide CoA is built up. This latter cyclizes to olivetolic acid (OA) by a α+β barrel (DABB) protein, properly called olivetolic acid cyclase (OAC) [15]. Prenylation (through a geranyl diphosphate from the 2-*C*-methyl-D-erythritol 4-phosphate; DOXP = 1-deoxy-D-xylulose 5-phosphate (MEP/DOXP) pathway) of olivetolic acid yields cannabigerolic acid (CBGA) by CBGA synthase [12]. CBGA can then undergo oxidative cyclization to achieve CBDA, THCA and/or cannabichromenic acid (CBCA) compounds. A schematic pathway is depicted in Figure 1. In particular, CBDA light- and/or heat-induced decarboxylation provides cannabidiol, recently defined as “a pharmacologic agent of wondrous diversity, an absolute archetypal dirty drug” [16].

## 3. CBDA as Bioactive Compound

The beneficial properties potentially exerted by CBDA are obscured by the plethora of bioactive effects of its neutral derivative. In fact, it is broadly known that CBD is characterized by an extreme pharmacological versatility [17]. Briefly recalling some literature data, CBD exerts a singular antagonistic action, as well as at low levels, towards CB1 receptors, when THC is co-present, and a weak affinity with the same receptor, if present individually [18]. It has modulatory and attenuating activity against the adverse THC effects (e.g., anxiety, tachycardia, appetite, and sedation) [19,20]. The modulation exerted by CBD upon THC effects in the CB_1_R and its possible allosteric nature were recently explored [21]. CBD is an analgesic [22], and it was suggested for the management of inflammation and joint pain [23]. Furthermore, this non-psychoactive compound is a neuroprotective antioxidant, much more powerful than ascorbate and α-tocopherol [24,25]. It acts as a vanilloid 1 transient receptor potential (TRPV1) receptor agonist, similarly to capsaicin [26], not explaining COX inhibition or eliciting side effects. Moreover, CBD also inhibits anandamide absorption, and its hydrolysis [27]. CBD is an antiepileptic [28], an antiemetic [29], and it is cytotoxic towards breast cancer cells and many other types of cell lines, while being cytoprotective towards normal cells [30]. Furthermore, this portentous compound is an antagonist towards the tumour necrosis alpha factor in mouse models affected by rheumatoid arthritis, improves adenosine A2A receptors through the adenosine transporter, and prevents the accumulation of prions and neuronal toxic substances [31,32]. Lipid synthesis in sebocytes is also inhibited by CBD, which further produces apoptosis at high doses in acne models [33]. Other studies highlighted that CBD is a critical factor in successfully treating intractable neoplastic pain in patients who do not respond to opioids.

**Figure 2:**
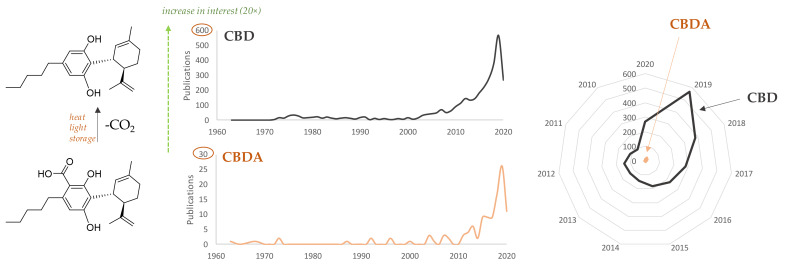
Number of papers dealing with the concept “CBD” or “CBDA” published in year range 1960–2020 (source: PubMed database, 18 April 2020). Data are plotted also through a radial chart that enhances, at one glance, the different interest in the two highly related compounds.

**Figure 3:**
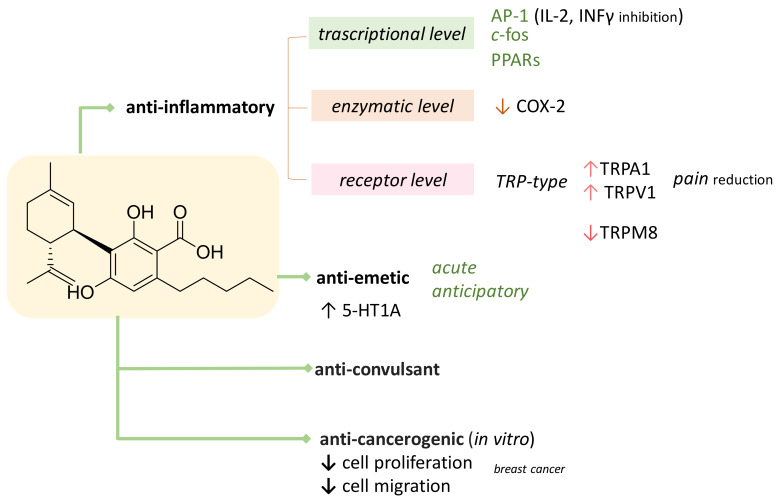
Overview of actual knowledge on CBDA bioactivity. COX-2, cyclooxygenase-2; TRPV1, vanilloid 1 transient receptor potential; TRPA1, ankyrin 1 TRP; AP-1, activator protein I.

The CBD bioactivity list could be even longer, and a detailed review would be really beyond our scope. It is certain that, to date, PubMed search of ‘cannabidiol’ as a key concept returns 2997 results, whereas only 104 outcomes are found for its carboxylated precursor (Figure 2). The first useful evidence dates back to just over a decade ago, when CBDA was found as a selective cyclooxygenase-2 inhibitory agent [34], and appeared to share with CBD the ability to activate vanilloid 1 and ankyrin 1 transient receptor potential (TRP) channels (TRPV1 and TRPA1, respectively), and to antagonize the Transient Receptor Potential Cation Channel Subfamily M Member 8 (TRPM8), a receptor activated during painful, inflammatory processes and in cold sensitization [35]. However, CBDA seems to exert these effects with significantly less potency than CBD. Figure 3 summarizes the actual knowledge on CBDA bioactivity.

In vivo studies, mainly carried out on rats, examined CBDA ability to inhibit vomiting induced by toxins or from movement (motion sickness) and to improve 5-HT1A receptors activation in the rat brain stem membrane. In fact, the anti-nausea effects of both CBD and CBDA are mediated by the action on the receptor. CBDA appears to be able to reduce emetic attacks and simultaneously increase the latency of the emesis onset in rats in response to movement with a more powerful effect than CBD [29,36]. CBDA reduces further anticipatory (conditioned) nausea, as well as by a 5-HT_1A_-dependent mechanism of action. This finding was in line to the synthesis of cannabidiolic acid methyl ester (HU-580), whose chemical features, slightly different from its natural precursor, seem to encompass CBDA chemical instability, and its susceptibility to decarboxylation [37]. Recent findings state that HU-580 also positively affects the sleep–wake cycle in male Wistar rats [38], and still CBDA anti-nociceptive activity was enhanced following methyl ester group addition [39].

The potential CBDA anti-inflammatory activity continues to be the main focus for deepened investigations. In this context, studies based on systemic or oral administration before and/or after the inflammatory and irritating carrageenan were carried out [40]. In particular, it was evidenced that CBDA at 10 μg/kg exerted an anti-inflammatory action when administered intraperitoneally 60 min prior to carrageenan, whereas in rodents pre-treated with 100 μg/kg, delivered by oral gavage, carrageenan-induced hyperalgesia favorably decreased [40]. CBDA, intraperitoneally administered at 0.1 μg·kg^−1^ dose, also showed anxiolytic-like effects under conditions of high stress [41]. The brain and plasma pharmacokinetic profile of CBDA, as well as of other acidic cannabinoids, has been recently defined; the rapid absorption at plasma level and the extremely low brain/plasma ratio were positively modulated when CBDA was administered in an alternate Tween 80-based vehicle. The anticonvulsant in a mouse model of Dravet syndrome is also reported [42]. PPARγ, a nuclear receptor expressed in several tissues and cell types, involved in inflammation and neurodegeneration, was found to be activated in 293T cells transfected with a pair of GAL4-PPARγ/GAL4-luc plasmids by CBDA in a more efficacious way in respect to CBD at high concentrations [43].

The CBDA anticancer activity was also preliminarily investigated on CEM (acute lymphocytic leukemia) and HL60 (promyelocytic leukemia) cells. The effect of CBDA, beyond other cannabinoids, was evaluated on cell viability, cell proliferation, and cell-cycle dynamics [44]. Data from these experiments, as well as those from MTT assay on human prostate carcinoma androgen receptor-positive (LNCaP) cells [45], evidenced that CBDA was less active than CBD. Therefore, no further attention was reserved to the potential anticancer activity of CBDA until it was tested towards MDA-MB-231 cells, a highly aggressive triple-negative breast cancer cell line. In particular, it was found to be able to inhibit breast cancer cells migration, and to downregulate the proto-oncogene *c*-fos and the cyclooxygenase-2 (COX-2) [46,47,48]. CBDA, at a concentration of 5 μM, seems to suppress COX-2 expression, and this activity was thought to be owing its ability to interfere with activator protein I (AP-I) activity. It has only more recently been observed that CBDA enhanced PPARβ/δ antagonist-mediated inhibition of COX-2 expression. In fact, PPARβ/δ, a member of the nuclear receptor (NR) superfamily, also expressed in the MDA-MB-231 cells, underwent CBDA-induced attenuation of its transcriptional activities [49].

Recent in silico study was carried out to investigate drug-like properties of CBDA and other phytocannabinoids [50]. Molecular properties, such as number of hydrogen bond acceptor (HBA) and hydrogen bond donor (HBD), partition coefficient (cLogP), polar surface area (PSA), and the number of rotatable bonds (NROTB), were calculated using Molinspiration Cheminformatics software (Table 1). CBDA molecular structure, obtained in the form of Simplified Molecular-Input Line-Entry System (SMILE), was imported using PubChem Compound. CBDA’s topological PSA (TPSA) was in line with an absorptivity of more than 90%. Furthermore, when drug likeness score for G protein-coupled receptors (GPCRs) ligands, ion channel modulators, kinase inhibitors, nuclear receptor ligands, and protease inhibitors were predicted, it was observed that CBDA is moderately active in all bioactive scores [50]. Unfortunately, the more recent drug discovery approach for in silico pharmacokinetic profile did not consider CBDA among the compounds investigated to get structural insights into the selection of cannabinoid scaffolds for the development of antitumor drugs [51].

The available data clearly show that we are dealing with a molecule whose bioactivity and pharmacological power, probably owing to the strange past of the source plant, has not been properly and thoroughly investigated. A single article described CBDA’s selectivity for the COX-1 enzyme, opening to a contradiction that needed to be explored [52]; however, this did not happen. Thus, optimizing its full exploitation and deepening the knowledge of its pharmaceutical efficacy should be pursued. This could be auspiciously achieved considering the high presence (in some cases, the abundance) of this compound in the different parts of industrial hemp plants.

## 4. CBDA: The King Compound in Industrial Hemp and Its (By)-Products

In recent years, Italy, as other European countries, was hit by the industrial hemp revolution. The food sector, more than others, has acquired the benefits of hemp cultivation, launching on the market products mostly deriving from the processing of hemp seed. Concurrently, the fear of the presence of possible contamination of edible products with cannabinoids, instead present in other parts of the plant, has given way to a whole series of analyses, also through the use of the most advanced techniques, to define the presence, the relative abundance, and the origin (e.g., leaf, florescence) of these substances during the processing [53,54,55]. Indeed, hemp seed oil contains, among its constituents, acidic cannabinoids, some of them highly oxygenated. Cannabidiolic acid is the most representative cannabinoid compound in hemp seed oil, and the CBDA/CBD ratio was proposed as a marker of storage conditions and production process [7]. Furthermore, the CBDA content could be particularly elevated in waste materials, as trimming materials or hemp pollen. Thus, based also on recent interest in the CBDA’s bioactivities, studies aimed at its isolation and purification have been carried out in order to obtain its high qualitative and quantitative "recovery". This need has prompted us to use multiple, combined, and alternative techniques that "simplify" the chemical composition of two different hemp-derived products, such as hemp seed oil and hemp pollen. Even in this case, there are few studies in the literature concerning the CBDA purification, where great attention is paid to the neutral analogue. It is noteworthy that identification studies in which liquid chromatography techniques coupled with DAD or MS detection are the masters [56,57], while there have been few real attempts to achieve the molecule’s purification.

Popp et al. [58] optimized the CBDA isolation procedures for the application of centrifugal partition chromatography (CPC—Fcpc (fast centrifugal partition chromatography) on an extract obtained by extraction with supercritical fluid (SEF); the fraction obtained by the CPC was further fractionated by liquid–liquid extraction. The use of centrifugal partition chromatography for cannabinoid isolation was previously reported [59]—the novelty of Popp’s study lies in the possibility of modulating the separation using the pH-zone-refining method. CPC is a counter-current liquid-liquid partitioning chromatographic technique in which the stationary phase is immobilized by centrifugal force, while the mobile phase is pumped at high flow rates. Sample components are divided between the mobile and stationary phases and are separated on the basis of the differences in their partition coefficients. CPC offers particular advantages in the isolation of compounds; there is no irreversible retention, it can cover a wide polarity scale, and has a very high capacity owing to the large stationary phase volume involved in the separation process. Another recent study by Brighenti et al. [54] did not approach the isolation, but used core-shell technology to optimize a chromatographic strategy for the separation of non-psychoactive phytocannabinoids in hemp extracts obtained with four different extraction procedures: ultrasound-assisted maceration, microwave-assisted extraction, maceration dynamics, and extraction with supercritical fluids. The advantages of core–shell technology lie in the fact that, compared with completely porous particles, the melt-core ones have a much shorter diffusion path owing to the solid core. This tends to reduce the axial dispersion of the solutes and minimize the peak widening. Looking back to the scientific literature of the 1970s, the existence of a manuscript entitled “Isolation of two constituents (cannabidiolic acid and tetrahydrocannabinolic acid) from *Cannabis sativa* L.” by preparative thin layer chromatography published was published in *Annales Pharmaceutiques Françaises* [60]. The manuscript, whose authors are Paris & el-Mounajjed, is in French and is not available online.

## 5. CBDA Isolation and Chemical Characterization from Hemp Pollen: Our New Goal

CBDA isolation and purification represent a prerogative of our latest laboratory activities. In particular, hemp pollen (HP), which is a resin produced by isolating the trichomes from the plant inflorescences by sieving process, was selected as CBDA source. HP was previously observed as a rich source of cannabinoids and flavonol glycosides [61], but GC-FID and GC-MS analyses, while highlighting the occurrence of 16 cannabinoids, were not able to analytically discriminate acidic from not-acidic compounds. Herein, hemp pollen, provided by Hemp Farm Lab farmers (Caserta, Italy), underwent ultrasound accelerated maceration (UAM) (Branson UltrasonicsTM Bransonic^TM^ M3800-E, Danbury, CT, USA). Exploiting the alternation of pressure and cavitation as useful means for cell decomposition and, therefore, for the release of the intracellular metabolic content, represented the starting point of our investigation. On the basis of the use of chloroform/*n*-hexane (1:1, *v/v*) as extracting solution, hemp pollen (10 g; drug/solvent ratio 1:5) underwent three extraction cycles (30 min each). A mixture enriched with cannabinoids was obtained (yield 26.5%).

**Figure 4:**
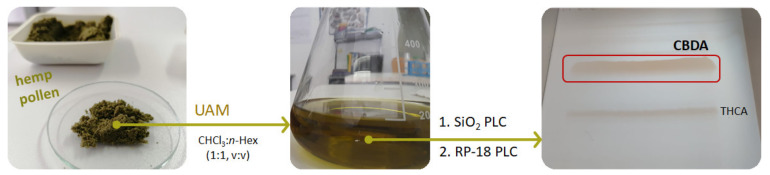
Hemp pollen underwent ultrasound accelerated maceration (UAM), and an aliquot of hemp pollen (HP) extract (60 mg) was then chromatographed by preparative thin-layer chromatography (PLC) using a precoated silica gel 60 F254 (20 × 20 cm, 1 mm, Merck, Darmstadt, Germany). The organic lower phase of a biphasic CHCl_3_/MeOH/H_2_O (13:7:7, *v/v/v*) solution served as mobile phase. Among the five fractions obtained, one was further fractionated by thin-layer chromatography using a precoated silica gel 60 RP-18 F254S (20 × 20 cm, Merck, Darmstadt, Germany). Elution was twice by MeCN/H_2_O (4:1, *v/v*) solution. The main compound (yield 45%) was identified, by means of spectroscopic and spectrometric techniques, as cannabidiolic acid, whereas the other compound was THCA-A.

The scheme of applied extraction and fractionation steps is depicted in Figure 4. The compound was unequivocally identified through spectrometric and spectroscopic analyses, able to strongly differentiate it from THCA-A constitutional isomer, equally purified through preparative thin-layer chromatography (PLC). Indeed, isolated compound was first analyzed by HPLC-UV-DAD.

**Figure 5:**
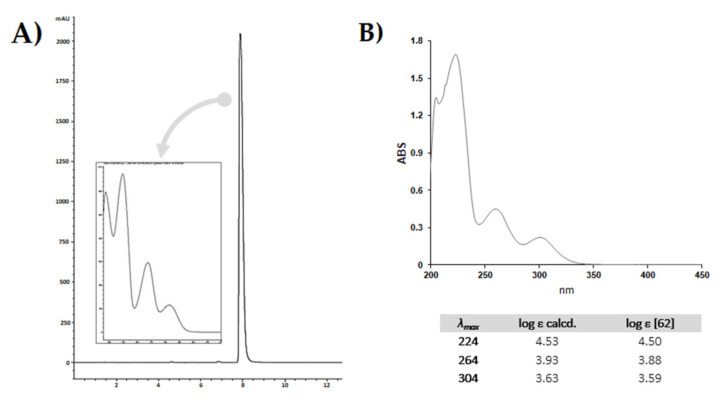
(**A**) HPLC-UV-DAD chromatographic profile of purified compound; UV-DAD spectrum is into the box. The RP-18 PLC-isolated compound (10 mg/mL, MeOH) was analysed by the HPLC 1260 INFINITY II system (Agilent, Santa Clara, CA, USA), equipped with an Agilent G7129A autosampler, an Agilent GY115A DAD-UV-visible detector, and a Quaternary pump Agilent G711A. The analysis was carried out using the Luna^®^ Phenyl-Hexyl column (150 × 2 mm, 3 µm). The mobile phase consisted of a binary solution A: 0.1% HCOOH in H_2_O, B: 0.1% HCOOH in CH_3_CN. A linear gradient was started at 55% B, held for 1.5 min, and linearly ramping to 95% B in 6.50 min. The mobile phase composition was maintained at 95% B for another 2 min, then returned to the starting conditions and allowed to re-equilibrate for 3 min. The total analysis time was 13.00 min. The injection volume was 3.0 μL; the flow was set at 0.3 mL/min. (**B**) UV-Vis spectrum of CBDA from hemp pollen, acquired in the range 190–450 nm using a UV-1700 double beam spectrophotometer (Shimadzu, Kyoto, Japan). The pure metabolite was solubilized in MeOH with a final concentration of 0.5 × 10^-5^ M. The table below shows the calculated molar extinction coefficients (log ε). Literature values are reported for comparison.

The chromatographic profile is shown in Figure 5A, together with the DAD spectrum, which is comparable to that reported in the literature [62]. In order to calculate the molar extinction coefficients at the three maxima absorption (λ_max_) exhibited by the compound, UV-Vis spectrophotometric analysis was carried out. The coefficients ε were in accordance with those previously reported in the literature (Figure 5B).

^1^H-NMR and ^13^C-NMR spectra were acquired and compared with literature data [63].

**Figure 6:**
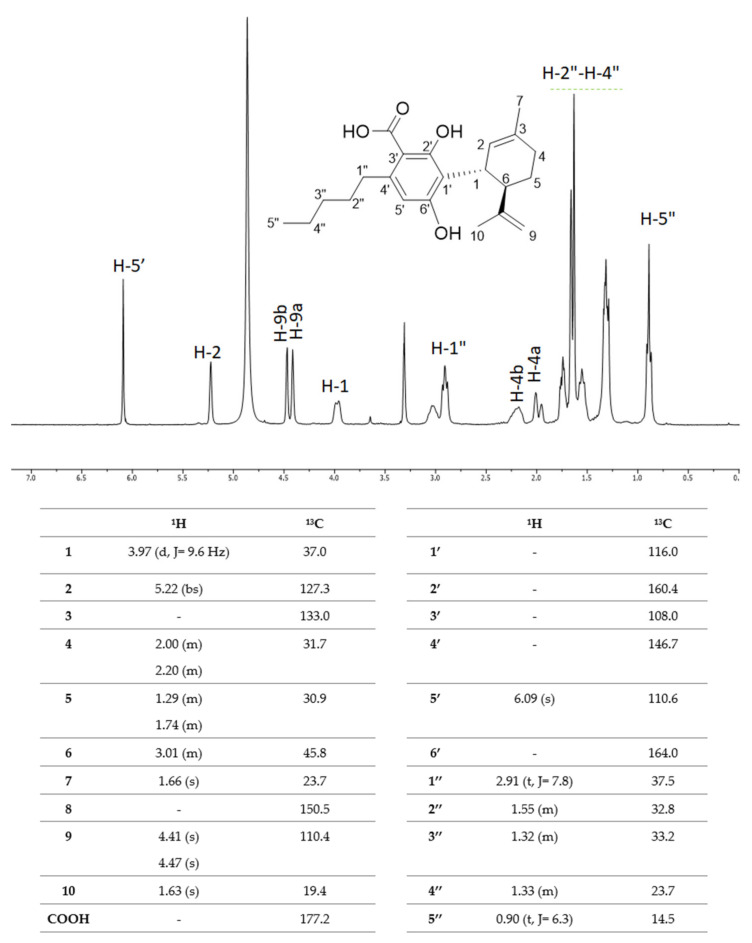
^1^H-NMR spectrum of CBDA. ^1^H-NMR and ^13^C-NMR assignments are tabulated below. NMR spectra were recorded at 300.03 MHz for ^1^H and 75 MHz for ^13^C on a Varian Mercury 300 spectrometer Fourier transform NMR (Varian, Palo Alto, CA, USA) in CD_3_OD at 25 °C. Chemical shifts are reported in δ (ppm) and referenced to the residual solvent. J (coupling constant) are given in Hz.

The ^1^H-NMR spectrum showed the pentyl chain H-1”-H-5” protons in the spectral region of 0.90–2.91 ppm (Figure 6). In particular, the triplet at δ_H_ 0.90, integrable for three protons, was attributable to H-5" protons, whereas the three multiplets at δ_H_ 1.32, 1.33, and 1.55 ppm were consistent with the methylene protons H-3", H-4", and H-2", respectively. H-1" benzyl protons were at δ_H_ 2.91. In the aliphatic region of the spectrum, the protons of the monoterpene core of CBDA were also distinguishable. In particular, the diastereotopic protons H-5 and H-4 resonated as multiplets at 1.29/1.74 ppm and 2.00/2.20 ppm, respectively, while the H-2 olefin proton was observed as a singlet at δ_H_ 5.22. The singlet at δ_H_ 1.63, integrable for three protons, was attributable to H-10 methyl protons of the propenyl chain, whereas the two olefin protons H-9a and H-9b were at δ_H_ 4.41 and 4.47, respectively. The de-shielding effect of the nearby aromatic ring and of the olefinic function C-2‒C-3 brought H-1 methine proton to resonate as a doublet at δ_H_ 3.97, whereas the neighboring H-6 proton was detected at δ_H_ 3.01. Finally, H-7 methyl protons were detectable at δ_H_ 1.66. In the aromatic region, H-5’ proton, which correlated with the carbon at δ_H_ 110.6 in the HSQC experiment (spectrum not shown), was evident at δ_H_ 6.09.

The presence of the carboxylic function was highlighted in the ^13^C-NMR experiment, which showed the relative carbon resonating at δ_C_ 177.2. The ^13^C-NMR data were in accordance with an aromatic nucleus, binding the CBDA monoterpene core, stabilized by the formation of a stable intramolecular hydrogen bond, which massively influenced the C-1’ resonance, bringing it to δ_C_ 116.0.

**Figure 7:**
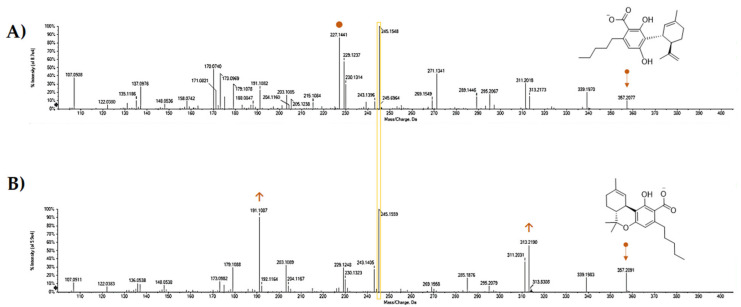
TOF-MS/MS spectra of CBDA (A) and Δ^9^-THCA (B). Both the compounds, reconstituted in methanol LC-MS grade, at a 10 mg/mL dose level, were analyzed using a Shimadzu NEXERA UHPLC system (Shimadzu, Tokyo, Japan) equipped with a Luna^®^ Omega Polar C18 column (1.6 μm, 50 × 2.1 mm i.d, Phenomenex, Torrance, CA, USA). The separation was achieved using a binary solution (**A**) H_2_O (0.1% HCOOH), (**B**) CH_3_CN (0.1% HCOOH) using a gradient program, which started at 25% B, which linearly ramped up to 55% B in 1 min, and then to 95% B in other 7 min, where it held for 1.0 min. Then, the initial condition was restored and held for another 2 min. The total run time was 11.5 min, with a flow rate of 0.4 mL min^−1^. The injection volume was 2.0 μL. MS analysis was performed using a hybrid Q-TOF MS instrument, the AB SCIEX Triple TOF^®^ 4600 (AB Sciex, Concord, ON, Canada), equipped with a DuoSprayTM ion source (consisting of both electrospray ionization (ESI) and atmospheric pressure chemical ionization (APCI) probes), which was operated in the negative ESI mode. The MS parameters were as follows: curtain gas (CUR) 35 psi, nebulizer gas (GS 1) 60 psi, heated gas (GS 2) 60 psi, ion spray voltage (ISVF) 4.5 kV, interface heater temperature (TEM) 600 °C, and declustering potential (DP) −70 V. In TOF-MS/MS experiments, collision energy (CE) applied was −45 V with a collision energy spread (CES) of 15 V, collision energy (CE) of −10. The instrument was controlled by Analyst^®^ TF 1.7 software (AB Sciex, Concord, ON, Canada, 2016), while data processing was carried out using PeakView^®^ software version 2.2 (AB Sciex, Concord, ON, Canada, 2016).

**Figure 8:**
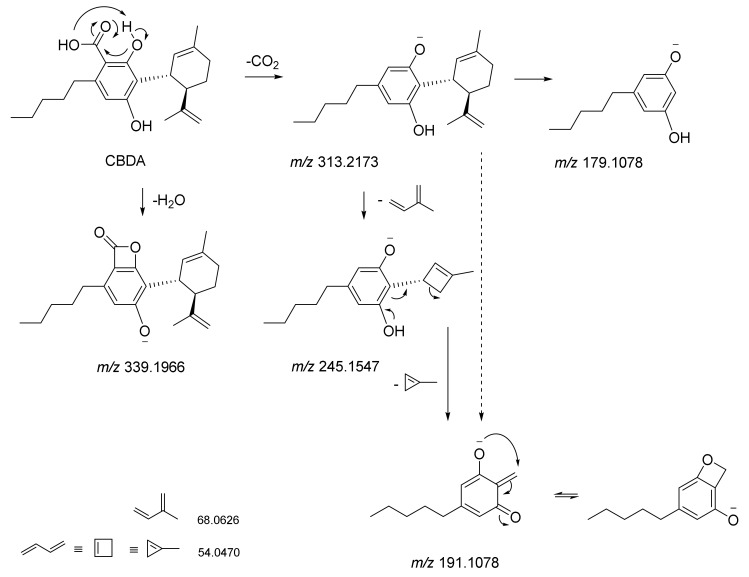
Proposed fragmentation pattern of CBDA compound based on TOF-MS/MS data.

Electrospray ionization (ESI)-QqTOF-MS/MS analysis appeared to be another tool able to discriminate the two constitutional isomers. It was recently stated that Δ^9^-THCA and CBDA exhibit MS/MS spectra similar to their respective neutral compounds, with additional neutral losses of CO_2_ and H_2_O because of decarboxylation and dehydration [64]. Indeed, some our analyses could further detail the MS/MS technique value. Both the molecules showed the [M-H]^-^ ion at *m/z* 357.2071, in accordance with the molecular formula C_22_H_30_O_4_. The TOF-MS/MS fragment ion at *m/z* 245.1548(59) as base peak, owing to −112 Da neutral loss. This latter could be the result of concurrent neutral losses of CO_2_ (−44 Da) and isoprene moiety (−68 Da). Dehydration and decarboxylation of the deprotonated molecular ion were hypothesized as fragmentation mechanisms that arise from the structural proximity of the phenolic functions with the carboxyl group. The relative abundance of the ion at *m/z* 191.1082(7) appeared to be one of the key fragments for discrimination purposes. In fact, this ion showed an intensity of 90.5% in THCA-A, whereas it was 24.6% in CBDA TOF-MS/MS spectrum (Figure 7 and Figure 8). Another diagnostic tool could be the ratio of the intensity of the ions deriving from dehydration and decarboxylation. In particular, the ratio between the ions at *m/z* 313.22 and 339.20 was 0.75 and 3.12 for CBDA and THCA-A, respectively. CBDA TOF-MS/MS spectrum also showed an abundant ion at *m/z* 227.1441, almost undetectable in the THCA-A TOF-MS/MS spectrum, which was owing to the loss of 130 Da (−68 + 62(44 + 18)) from the deprotonated molecular ion.

**Figure 9:**
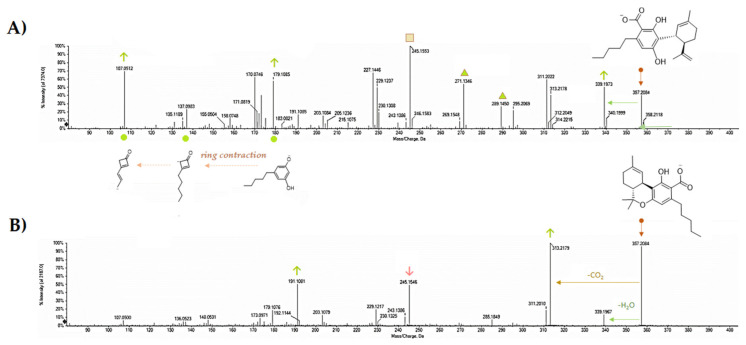
TOF-MS/MS spectra of CBDA (**A**) and Δ^9^-THCA (**B**) acquired using a hybrid Q-TOF MS instrument, the AB SCIEX Triple TOF^®^ 4600 (AB Sciex, Concord, ON, Canada) and MS parameters were as follows: curtain gas (CUR) 35 psi, nebulizer gas (GS 1) 60 psi, heated gas (GS 2) 60 psi, ion spray voltage (ISVF) 4.5 kV, interface heater temperature (TEM) 600 °C, declustering potential (DP) −75 V, and collision energy (CE) −5. In TOF-MS/MS experiments, collision energy (CE) applied was −55 V with a collision energy spread (CES) of 35.

The fragment ion at *m/z* 271.1341 (43.2%), which was recently suggested to derive from retro Diels–Alder reaction from the [M-H-H_2_O]^-^ ion, could also be considered a discriminant ion, and corresponded to the breakdown of the monocyclic monoterpene nucleus. The acquired data allow us to state that tandem mass spectrometry is useful for differentiating the two isomers, contrary to what was recently observed by Citti et al. [65], who claim that the distinction between CBDA and THCA can only be made by considering the different retention time. Moreover, to further strengthen this concept and to highlight its relativity in optimizing mass spectrometric analysis, some parameters were modified, among which collision energy and its spread, as well as declustering potential, which is the voltage applied to the orifice that helps to prevent the ions from clustering together. It appeared clear that parameters modification made Δ^9^-THCA-A more suitable to decarboxylation, whereas the characteristic loss of −112 Da was the main CBDA feature for establishing its most stable anion (Figure 9). Thus, no absolute assumption could be carried out. It is certain that the concurrent loss of CO_2_ and isoprene more favorably occur in CBDA than in Δ^9^-THCA-A, and that for qualification and quantification purposes, the ions at *m/z* 245, 179, and 107 should be considered for CBDA, whereas the ions at *m/z* 313 and 191 better recognize Δ^9^-THCA-A.

**Figure 10:**
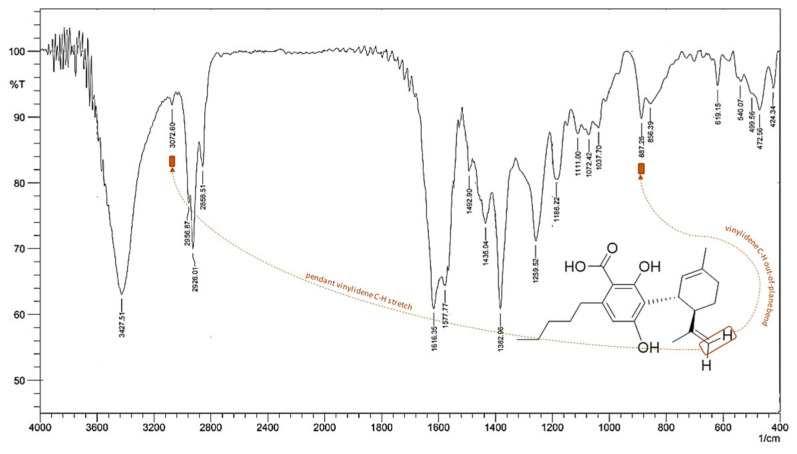
Fourier transform infrared spectroscopy (FT-IR) spectrum of CBDA, which was recorded in the region of 400–4000 cm^–1^ using the Prestige 21 system (Shimadzu, Japan), equipped with a DTGS KBr (tridiglycinsulfatedeuterate with KBr window; 8000–400 cm^−1^) detector, with a 4 cm^−1^ (45 scans) resolution. The disc having a diameter of 13 mm, a thickness of 2 mm, weight of 200 mg, and containing 1% by weight of sample in KBr, was obtained by pressing the sample powder in a cylindrical support using a manual Specac press. The spectral analysis was carried out using the Prestige software (IR solution).

Fourier transform infrared (FT-IR) spectroscopy was also used for characterizing CBDA [62], although a clear description of FT-IR data was not provided. Herein, FT-IR spectrum, acquired for the pure compound from hemp pollen, is reported (Figure 10). The 3427 cm^−1^ broad band was attributable to the stretching of the O–H bond of the carboxylic function, whereas the 3073 cm^−1^ band was ascribable to the stretching of the C–H bonds of the alkenyl and aromatic groups. The stretching of the carbonyl bond of the carboxylic function, which is strongly affected by the establishment of an intramolecular hydrogen bond with the vicinal hydroxy group, could be masked by the broad band at 1616 cm^−1^, attributed to the latter by the stretch C–C in the aromatic ring. The presence of the carboxylic function was also defined by the stretching of the C–O bond at 1259 cm^−1^.

The bending vibrations of the O–H bond at 1435 cm^−1^ seemed to be distinguished from the bending modes of the C–H bonds present in the same spectral region. The bending mode in the plane of the phenolic group was located at 1393 cm^−1^, while the bending vibration outside the plane was at 619 cm^-1^. The weak bands of overtones between 2200 and 1620 cm^−1^ and the bands ascribable to the C–C stretching vibrations in the aromatic ring were detectable at 1616, 1578, and 1493 cm^−1^. The bending vibrations in the plane of the aromatic C–C bonds were at 1111, 1072, and 1038 cm^−1^. The bending vibration outside the plane of the C–H bond at 887 cm^−1^ appeared strongly diagnostic as it could be attributable to the C=C vinylidene bond. Out-of-plane bending modes are typically informative of the location and geometry of the double bond, being able to discriminate terminal and median bonds. Finally, according to previous findings [66], when optical rotations were acquired using a PerkinElmer polarimeter (series 343; PerkinElmer Life and Analytical Sciences, Shelton, CT, USA), an [α]_d_^20^ –59.05 (c 0.127, MeOH) was found. Thus, isolated CBDA showed negative optical rotation, as the other CBD-type cannabinoids reported in literature, as well as those with different lengths of the alkyl chain. In particular, it was reported that absolute configuration of CBD-type cannabinoids is (−)-*trans*-(*1R*,*6R*) [66].

## 6. Conclusions

Cannabidiolic acid is an understudied compound right now. Its pharmaceutical and nutraceutical enforceability is still far from being achieved, but the high content of this compound in hemp varieties, cultivated for food purposes, suggests the need to further deepen knowledge of its chemical and biological features, as well as to fully and well exploit it. It is reasonable to state its content is massive in hemp wastes, such as pollen. In fact, the recovery of pollen from industrial hemp cultivars could represent an abundant source of non-psychoactive acid phytocannabinoids, of which cannabidiolic acid represents the main molecule. The use of "primitive" chromatographic techniques can represent, downstream of an alternative extractive approach with low environmental impact, an effective tool for obtaining the compound in good yield. In fact, the isolation and purification of the CBDA were carried out, starting from an extract enriched in phytocannabinoids by adopting "relatively" simple, fast, and inexpensive techniques such as direct and/or reverse phase thin layer chromatography. The relative abundance of this compound in hemp-based foods requires an effort by the scientific community to fully understand its action, in the presence and absence of similar lipophilic constituents such as the polyunsaturated fatty acids of which hemp oil is rich. Furthermore, the purification takes place, using it in the same way as expensive commercial standards, as an aid to the qualitative and quantitative analyzes aimed at quantifying its content in the various matrices obtained in the different stages of hemp processing, from cultivation to the formulation of food for human and animal consumption. Indeed, although the nutraceutical potential of this compound has not yet been defined, it is possible to hypothesize that its presence in hemp seed-based food products could provide benefits that go far beyond the known hemp seed high nutritional value. Moreover, the recovery of the compound from not-edible hemp parts could take advantage from its use as bioactive. It appears clear that there is still much to investigate, and other great efforts should be pursued. The main goal of this review was to pool actual CBDA knowledge together, for going on to acquire day by day new insights and new perspectives of bioanalysis for its future exploitation.

## Figures and Tables

**Table 1:** Computed physicochemical properties and bioactivity scores of cannabidiolic acid (CBDA). HBD, hydrogen bond donor; HBA, hydrogen bond acceptor; cLogP, partition coefficient; TPSA, topological polar surface area; NRTOB, number of rotatable bonds; GPCR, G protein-coupled receptor.

*Canonical smile* CCCCCC1=CC(=C(C(=C1C(=O)O)O)C2C=C(CCC2C(=C)C)C)O					
MW (≤500)*	HBA (≤10)*	HBD (≤5)*	cLogP (≤5)*	TPSA, A^2^ (≤140*; ≤60#)	NRTOB (≤10)*
358.48	4	3	6.43	77.75	7
Good oral absorption*, Completely absorbed^#^					
*GPCR ligand*	*Ion channel modulator*	*Kinase inhibitor*	*Nuclear receptor ligand*	*Protease inhibitor*	*Enzyme inhibitor*
−0.39	−0.05	−0.74	−0.30	−0.63	−0.09
